# Shedding light on the structural properties of lipid bilayers using molecular dynamics simulation: a review study

**DOI:** 10.1039/c8ra08441f

**Published:** 2019-02-06

**Authors:** Sajad Moradi, Amin Nowroozi, Mohsen Shahlaei

**Affiliations:** Nano Drug Delivery Research Center, Kermanshah University of Medical Sciences Kermanshah Iran; Pharmaceutical Sciences Research Center, Faculty of Pharmacy, Kermanshah University of Medical Sciences Kermanshah Iran; Medical Biology Research Center, Kermanshah University of Medical Sciences Kermanshah Iran mohsenshahlaei@yahoo.com m.shahlaei@kums.ac.ir

## Abstract

In recent years, a massive increase has been observed in the number of published articles describing accurate and reliable molecular dynamics simulations of lipid bilayers. This is due to several reasons, including the development of fast and efficient methods for treating long-range electrostatic interactions, significant progress in computer hardware, progress in atomistic simulation algorithms and the development of well-validated empirical molecular mechanical force fields. Although molecular dynamics is an effective approach for investigating different aspects of lipid bilayers, to the best of our knowledge, there is no review in the literature that explains the different analyses that can be carried out with membrane simulation. This review gives an overview about the some of the most important possible analyses, technical challenges, and existing protocols that can be performed on the biological membrane by molecular dynamics simulation. The reviewed analyses include the degree of membrane disruption, average area per lipid, probability distributions for the area per lipid molecule, membrane thickness, membrane area compressibility, lateral diffusion, rotational diffusion, order parameters, head group tilt, electron density profile, mass density profile, electrostatic potential profile, ordering of vicinity waters, number of hydrogen bonds, and radial distribution function.

## Abstract 2

This review gives an overview about the some of the most important possible analyzes, technical challenges, and existing protocols that can be performed on the biological membrane by the molecular dynamics simulation.

## Graphical abstract

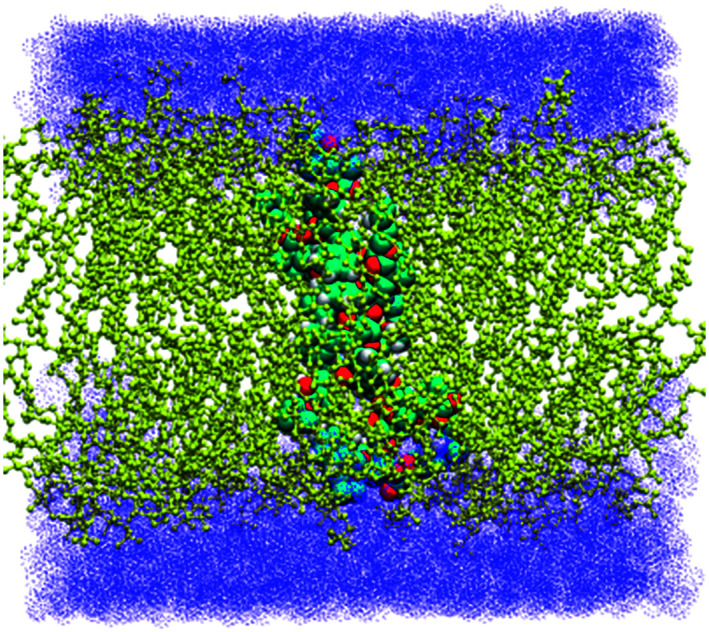

## 1 Introduction

Biological lipid bilayers have a multitude of biological roles, such as facilitating the synergy between diverse lipids, proteins, peptides, and carbohydrates.^1^ The description of the structure, functions and things that may happen to the lipid bilayers when the membrane is exposed to environmental changes or interact with a molecule at the atomistic scale, it can be daunting, even for experienced researchers. With the availability of powerful hardware and advanced algorithms in the scientific community, new effective methods for verifying theoretical results have been developed for complex biological systems. These new tools have also allowed the simulation of complex phenomena in the condensed matter field.^2,3^ Together, this has led to the era of “computer experiments”. Particularly, molecular dynamics (MD) simulation methods were developed by Alder and Wainwright^4^ in 1957 to simulate the behavior of hard spheres in a box depending on temperature and density. Generally, the molecular dynamics simulation method is a greatly appreciated tool for obtaining structural and dynamical data on different systems as complicated as lipid membranes. Although theoretical approaches lose their power owing to oversimplifying assumptions, and experimental results may scatter widely and are often difficult to interpret on atomistic details, the molecular dynamics simulation technique provides trajectories that are relatively simple and straightforward to interpret.^5^ Furthermore, the molecular dynamics simulation technique can provide relevant details that are experimentally inaccessible or expensive and time consuming.^6^ Arguably, one of the important goals in the molecular dynamics simulation of molecular systems is sampling of the phase space accessible to the system. The phase space can be defined as the set of positions and linear momenta of the atoms belonging to the system in a given time interval. After the simulation is performed, statistical mechanical methods are employed to derive quantities that can be compared with the corresponding experimental results. Also, the results of statistical mechanics analysis can be used for the detailed study of the system.^7^ Although most of these analysis tools are well-known and standard for molecular systems, special tools have been developed for biological membranes.

A large number of structural, biological, and dynamic properties and phenomena of biological membranes have been studied by MD simulation methods. For example, Pandit *et al.* proposed an algorithm for the general description of rugged molecular scale interfacial surfaces and used it for the description of a phospholipid membrane/water interface.^8^ As another example, Saiz and Klein reported the structure of a fully hydrated mixed (saturated/polyunsaturated) chain lipid bilayer in the biologically relevant liquid crystalline phase using MD simulation.^9^ There are also numerous published papers about the MD simulations of membrane proteins.^10–14^ Böckmann *et al.* studied the influence of sodium chloride on a pure palmitoyl pleoyl glycerol phosphocholine (POPC) lipid bilayer *via* fluorescence correlation spectroscopy experiments and MD simulations.^15^ Lindhal and Edholm studied the structural fluctuations present in fully hydrated dipalmitoyl phosphatidyl choline (DPPC) bilayers using MD simulation. Also, the interaction of ions with membranes was investigated. For example, Gambu and Roux investigated the interaction of a potassium ion with a dimyristoylphosphatidylcholine (DMPC) bilayer membrane using MD simulation.^16^ Sachs and Woolf performed a set of all-atom molecular dynamics simulations to study the critical phenomena associated with the Hofmeister series of anions and lipid bilayers.^17^ Marrink *et al.* studied and reported the self-assembly of a lipid bilayer from an initial random dispersion of lipid molecules.^18^ The examples discussed above show that by using molecular dynamics simulations, atomic-level insights can be obtained into a growing variety of biological membrane systems of increasing size and complexity.^19^

However, there are a number of limitations to study the biological properties of membranes. The first limitation is the size of the system. Due to the computational limitations of current computers, a typical MD simulation of a biological membrane, mainly in each direction of the simulation box is about 500 Å or less. This only makes it possible to study the behavior of a limited number of membrane-forming molecules. Another serious limitation to be considered is the simulation time scale. Although, currently, the simulations of membrane phenomena have become larger and longer, reaching the scale of micrometers and microseconds, they are still several orders of magnitude far from the time and/or length scales of the most complex biological events.^20^

For example, the process by which lipid vesicles bud from the plasma membrane of cells involves length scales of about 100 nm and time scales of milliseconds or more. As another example, the motions in membranes range from conformational transitions of the lipid hydrocarbon tails on picosecond scales to bending of 10 μm-sized patches extending to several milliseconds. Due to these mentioned limitations, it is imperative to have analysis methods that can study phenomena and extract the required information.^20^

Another area in which molecular dynamics can play an important role is the study of the behavior of intrinsically disordered proteins (IDPs) in model membranes since it is very difficult to crystalize and prepare protein XRD patterns in real lipid membranes. Also experimental results have shown that many molecular aspects of cellular phenomena such as protein folding, protein–protein and protein–membrane interactions occur within a very short time of nano and/or microseconds. Thus, information related to the early stages of these critical times is not fully accessible or is very hard and expensive to obtain experimentally. Therefore, undoubtedly, fast and inexpensive molecular modeling and simulation techniques can play an indispensable role in providing atomic details of the first steps of IDS and membranes.^21–24^

Also an intrinsic biological phenomenon that can be analyzed in atomic details by MD simulation is fibrillated amyloid-membrane bio-interaction. Experimental methods have demonstrated the penetration and production of ion channels in membranes by amyloids.^25^ The good review written by Dong *et al.* fully demonstrated the computational methods used for the investigation the islet amyloid polypeptide-membrane bio-interaction.^26^

The present review gives an overview and discussion about the some of the most important possible analyses, technical challenges, and existing protocols that can be performed on the biological membrane by molecular dynamics simulation. The reviewed analyses include degree of membrane disruption, average area per lipid, probability distributions for the area per lipid molecule, membrane thickness, membrane area compressibility, lateral diffusion, rotational diffusion, order parameters, head group tilt, electron density profile, mass density profile, electrostatic potential profile, ordering of vicinity waters, number of hydrogen bonds, radial distribution function, and head group dynamics.

This review is organized as follows: in the next section, the basic theory of MD simulation of biological membranes will be described, and the software packages available for membrane MD simulation are reviewed. In the main section, the various MD simulation analyses that can be used for lipid bilayers are discussed.

## 2 Experimental and methods

### 2.1 MD simulation software and force fields

As an outstanding method in computational biophysics and biochemistry, MD simulation is a used technique to study the time dependent behavior of a molecular system (physical movements of atoms and molecules in the studied system).

The extensive use of MD simulations in chemical, toxicological and biochemical research is mainly due to the availability of effective and efficient software packages and the significant progress in the hardware and algorithms and growing computational power. A large number of software packages have been introduced for MD simulations based on algorithms that are compatible with highly complicated systems such as biological systems. Most of the software available have different tools and a comprehensive range of functions for analyzing the trajectory file(s) generated in a typical simulation. It should be notes that these software packages may have differences in the analytical tools, algorithms used, the force fields that they support, and accept file formats of structure and trajectory that were originally developed in other packages or studies (Table 1).

**Table 1:** Popular software packages for MD simulations

Software name	Interface	License	Reference
AMBER	CLI	Proprietary	18
CHARMM	CLI and optional GUI	Proprietary	19
Desmond	GUI	Academic	20
Gromacs	CLI	Open source	21
NAMD	GUI	Academic	22

In molecular modeling, the force field (FF) refers to a series of functions and parameters needed to calculate the energy potential levels of a system.^27^ The parameters used, *e.g.* mass and atomic volume, atomic charges, equilibrium and energy constants for bands, angles and dihedrals, may be obtained using experimental methods or *ab initio* calculations. Depending on the functions used and the method of obtaining the parameters, as well as the different atom types that can be calculated, different force fields are used to perform calculations on a specific category of materials and molecules. The various types of force fields based on the use in calculations of biological molecules are given in Table 2.^28–38^

**Table 2:** List and details of some force field methods used in molecular dynamics simulation

Name	Description	
Universal force filed (UFF)	The force field parameters are estimated using general rules based only on the element, its hybridization, and its connectivity, developed at Colorado State University	24
MARTINI	A coarse-grained FF developed at the University of Groningen by Marrink and coworkers, initially developed for molecular dynamics simulations of lipids	25
MM2, MM3, MM4	These FFs were developed by Norman Allinger, mainly for conformational analysis of hydrocarbons and other small organic molecules	26–28
Consistent force field (CFF)	Developed as a general method for unifying studies of energies, structures and vibration of general molecules and molecular crystals	29
Chemistry at HARvard molecular mechanics (CHARMM)	Set of force fields for molecular dynamics of lipids, proteins, acid nucleic and small organic compounds	30
GROningen MOlecular simulation (GROMOS)	A general-purpose molecular dynamics computer simulation package for the study of biomolecular systems	31
Assisted model building and energy refinement (AMBER)	A family of force fields for molecular dynamics of biomolecules originally developed by Peter Kollman's group at the University of California, San Francisco	32
COSMOS-NMR	Hybrid QM/MM force field adapted to a variety of inorganic compounds, organic compounds and biological macromolecules, including semi-empirical calculation of atomic charges and NMR properties	33
Condensed-phase optimized molecular potentials for atomistic simulation studies (COMPASS)	Parameterized for a variety of molecules in the condensed phase	34

### 2.2 Reliability of MD simulations

Theoretically all molecular systems such as macromolecules can be studied by MD simulations. It is critical that the MD simulation results be compared with experimental data to evaluate their reliability. The comparison between experimental and computational results measures the molecular modeling quality with respect to ‘reality’. If the agreement between the theoretical and experimental methods is sufficient, the MD simulation results present an atomistic structural interpretation of the obtained experimental information.^39^

A large number of experimental methods have been used to study the various properties of lipid bilayers at the molecular level. X-ray crystallography is used to study how lipid molecules are placed together and how these molecules pack against one another in a highly ordered environment.^40–42^ Water penetration in a lipid bilayer can be investigated *via* the neutron refraction method. In this method for determining the penetration of water into the lipid medium, the difference between the structure factors obtained in D_2_O and H_2_O is compared.^43,44^ The membrane thickness is checked *via* X-ray crystallography.^45^ Nuclear magnetic resonance (NMR) is used to obtain both structural and dynamic information regarding the fatty acid chains.^46,47^ NMR is also used for determining the number of gauche kinks present in the lipid bilayer, and thus can be used for determining the phase of the bilayer.^48,49^ Several florescence methods are used frequently to measure the lateral diffusion of bilayer lipids. These methods are based on the fluorescence from the molecules of interest or from light scattered from particles attached to single or small groups of membrane lipids or proteins. Fluorescence recovery after photobleaching (FRAP), fluorescence correlation spectroscopy (FCS) and single particle tracking (SPT) are presented respectively below.^50^

### 2.3 Protocol of MD simulations of lipid bilayer

A molecular dynamics simulation determines the phase space trajectories of membrane atoms by numerical integration of the Newtonian equations.^51^ The construction of such a complex system requires careful attention to several details to obtain a meaningful trajectory. Since the simulations are computationally very intensive, it is desirable to build a starting configuration that is as representative of the solvated protein–membrane system as possible, thereby limiting the required equilibration time.

The initial structure of the membrane can be prepared in two ways. The first method is to use a pre-prepared membrane that has reached equilibrium. In the second method, the membrane can be prepared using standard protocols. The initial structures are created by placing equal numbers of lipid molecules from an earlier simulation in two layers with a defined separation (for example 3 nm), using periodic boundary conditions. The position of each lipid is defined from the position of the carbon connecting the tails to the head group and the orientation by the average of vectors along the two tails. The lipids are randomly rotated, tilted by sufficient degrees (for example 30 degrees), and given enough spread (for example 0.3 nm) in the *z*-coordinate.^52^ A slab of bulk water with the same surface area as the monolayers is placed on each side of the bilayer. After constructing the simulation system, it should energy minimized for enough steps using an appropriate algorithm such as the steepest descent method.^53^

Conventionally, the membrane normal is oriented along the *z*-axis, and the center of the bilayer is at *z* = 0.

The time scale of the MD simulation of membrane systems depends on the phenomenon being studied, and therefore should not be too short. A short simulation time prevents the exact and accurate study of many membrane properties, *e.g.*, of phase transition, which occurs on a time scale in the range of microseconds or longer.

One of the most important advantages of the united atoms force field is use of nonpolar CH_2_/CH_3_ groups in the hydrocarbon tails, which results in a reduction in the number of atoms per lipid molecules.

Simulations of membrane systems can be done using different ensembles, for example constant volume (NVT),^54,55^ constant surface tension equivalent to a constant anisotropic pressure (NyT), and constant isotropic pressure (NPT).

Actually, constant volume means to keep the dimensions of a box constant, which is the standard condition to simulate a protein in a crystal lattice. However, this condition is not suitable for a lipid bilayer because the dimensions of the box are determined by the area and the length per lipid, which are not well known. Therefore, constant pressure is more suitable, but the pressure may be anisotropic.

The pressure is scaled to 1 bar separately in all three coordinate directions with a finite time constant (for example 0.5 ps).^56^ Application of pressure in all three coordinate directions results in zero average surface tension. Since the coupling time constant is, finite there are still significant fluctuations in pressure and surface tension, but when averaged over several nanoseconds, these are negligible. The average temperature of the membrane simulation system is set to a temperature above the gel-liquid phase transition because the biological membrane liquid phase is the main phase.

Numerous experimental and computational studies have been published on model systems consisting of a single lipid-like POPC.^57^ Such studies of simplified systems are essential and have increased the understanding of the properties of biological membranes. Also, more complicated systems have been studied, including more complicated and realistic systems consisting of mixtures of different molecules such as cholesterol.^58^

If we want to put a protein or any other molecule inside the membrane, the general strategy for creating a representative starting configuration for the system consists of randomly selecting lipids from a preequilibrated and prehydrated set, dispersing them around the protein, and reducing the number of core–core overlaps between the heavy atoms through systematic rigid-body rotations (for example around the *z*-axis) and translations (in the *xy* plane) of the preequilibrated and prehydrated lipid molecules.

## 3 Analysis

### 3.1 Degree of membrane disruption

Membrane disruption may occur by the interaction of various molecules (or ions) or/and because of severe changes in environmental conditions such as temperature or pressure. One of the best ways to gain enough information about the degree of membrane disruption is the amount of water molecules present across the membrane, which is called “degree of membrane disruption”. As the number of water molecules in the membrane becomes larger, the degree of membrane disruption is increased. In other words, there is a positive correlation between the number of water molecules present across the membrane bilayer and the degree of membrane disruption. The best way for calculation is to count the water molecules between the oxygens of the lipid chains averaged over the equilibrium time of the simulations.

### 3.2 Average area per lipid

One of the main parameters in describing membranes is the average area per lipid 〈*A*〉. The average area per lipid allows the determination of a measure for tuning the force fields and other parameters for membrane systems.^59^ Also, 〈*A*〉 is related to some lipid membrane properties, such as acyl chain ordering, compressibility, and molecular packaging, among others.^60^ This is one of the main objectives of the quantitative description of membranes using molecular dynamics simulations. Furthermore, *in vitro* studies of integral membrane proteins or peptides frequently require them to be reconstituted with membrane lipids,^10,11,13,14,61–67^ where the bilayer structural dimensions involving hydrophobic matching and area per lipid are important parameters.^68–70^

In many published research, if molecular dynamics simulation was carried out under NPT, because of pressure coupling, the box dimensions of the simulation was permitted to fluctuate. Therefore, the average area per lipid was calculated using the size of the simulation box in the *x*–*y* plane.

Usually, the time evolution of the average area per lipid during molecular dynamics simulation is followed and plotted. This parameter fluctuates around an average value. System convergence is determined visually by the flatness of the plot of area per lipid during the simulation time. The average area per lipid does not change noticeably from its initial value if a pre-equilibrated membrane is used. Thus, the bilayer is stable over the entire course of the simulation. There are several studies that showed the time-dependent area per lipid is also a good criterion to determine if the system has reached the steady state.^60,71^

**Fig. 1:**
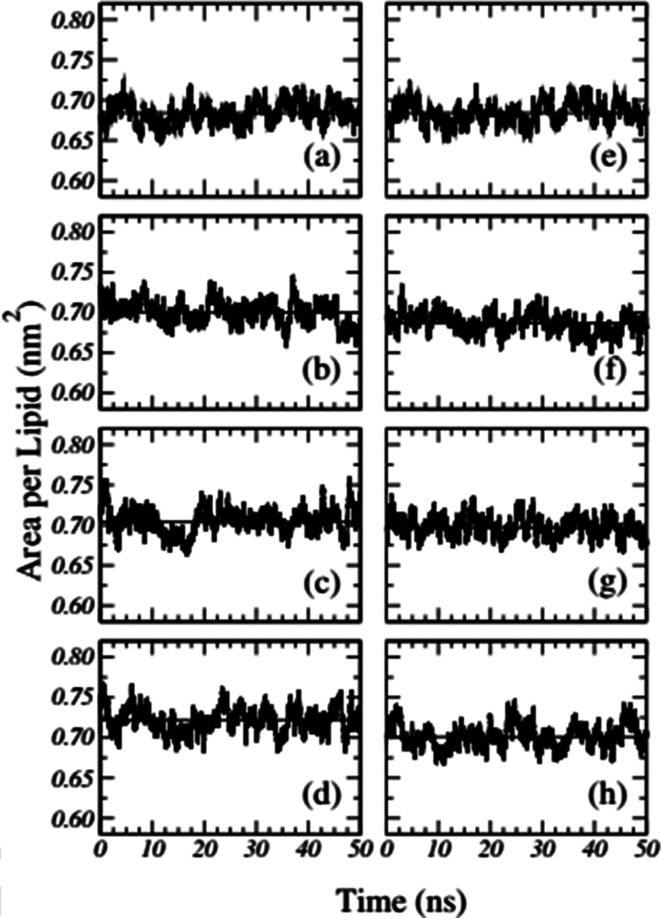
Time-dependent area per lipid in the presence of GLYP2 (left column) and GLYP (right column) at different G : L ratios of (a) and (e) 0 : 72; (b) and (f) 1 : 18; (c) and (g) 1 : 9 and (d) and (h) 1 : 3. Horizontal solid lines represent the mean average value of the area per lipid for the last 40 ns of the simulation. Reproduced from ref. 68 with permission from Elsevier.

Frigini and coworkers used MD simulations to study the effect of glyphosate (in its neutral and charged forms, GLYP and GLYP2–, respectively) on a fully hydrated DPPC lipid bilayer. The time evolution of the area per lipid resulting from unbiased simulations in their work is presented in Fig. 1. For the case of charged glyphosate (left column) and neutral glyphosate (right column) at different G : L ratios, the figures depicting the absence of herbicide, Fig. 1(a) and (e), are the same, which were duplicated to further clarify the comparison of the different systems and concentrations. As these results suggest, all the systems appeared to be stable after 5–10 ns of simulation.^72^

### 3.3 Probability distributions for the area per lipid

**Fig. 2:**
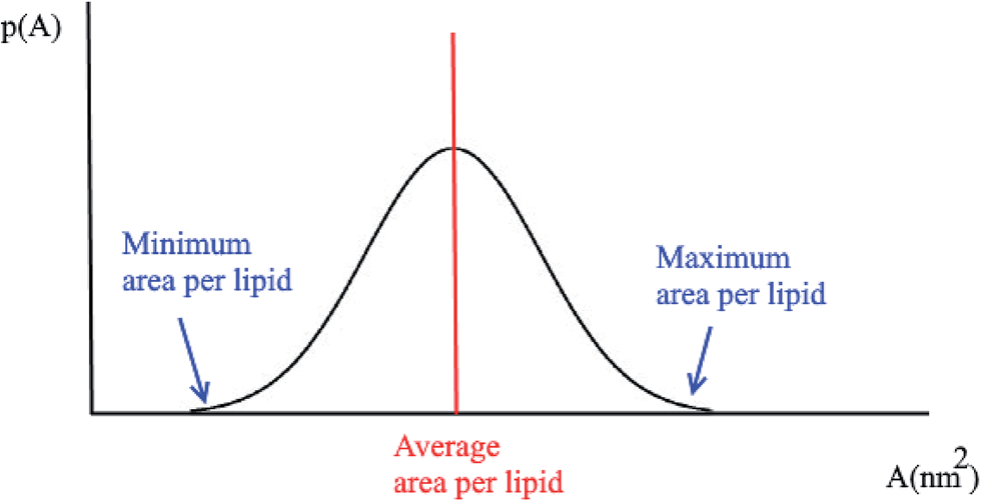
Typical probability distribution for the area per lipid.

Another quantity that is considered in the study of membranes and is of interest for a number of processes in lipid bilayers, *e.g.* the lateral diffusion of lipids in the bilayer plane, is the probability distributions for the area per lipid molecule, *P*(*A*). Usually this quantity follows a normal distribution. Using this plot, the minimum and the maximum area per lipid can be achieved (Fig. 2). Also, the median of this distribution represents the average area per lipid during the simulation.

### 3.4 Membrane thickness

**Fig. 3:**
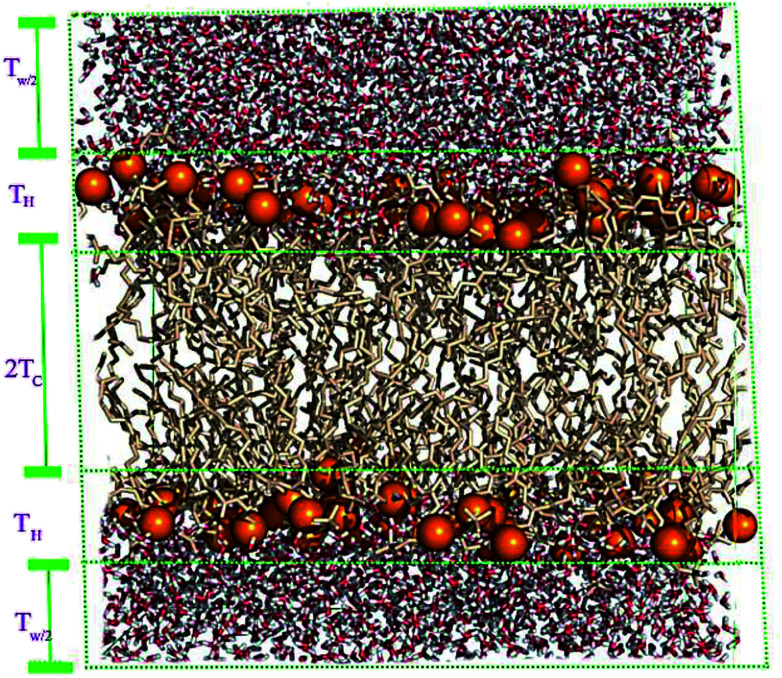
Schematic depiction of the multilamellar structure of the lipid membrane. In this multilamellar structure, *T* = *T*_W_ + *T*_B_ is the sum of the interlamellar water thickness and *T*_W_ = 2*T*_W/2_ and the membrane thickness *T*_M_ = 2(*T*_H_ + *T*_HC_). Here *T*_HC_ is the hydrocarbon thickness per membrane leaflet and *T*_H_ is the head group layer thickness.

A membrane forms a very oriented multilamellar structure (Fig. 3). A multilamellar structure is formed after hydration due to the amphiphilic nature of the membrane. This amphiphilic nature comes from hydrophobic characteristics and van der Waals forces.^73^ As can be seen in Fig. 3, water molecules surround the membrane polar head groups^74^ and partially penetrate the membrane,^75^ which called interlamellar water. The total thickness of the interlamellar water in both sides of the bilayer is denoted by *T*_W_. Therefore, *T*_W/2_ is the half-water thickness on either side of the membrane. Also, the thickness of the membrane polar head groups is denoted by *T*_H_. This layer is faced toward the interlamellar water.

The membrane thickness is defined as the distance between the average positions of the lipid phosphate groups (*T*_M_ = 2(*T*_H_ + *T*_HC_)). This quantity can be computed from the total electron density profile.

In a typical molecular dynamics simulation, to calculate membrane thickness, only phosphorus atoms are considered. The mass density profile of phosphorus atoms during molecular dynamics simulation is calculated along the bilayer and the normal distance between the peaks in the generated plot is thickness. This approach provides an average P–P distance, which can be used to describe the bilayer thickness. Another way to calculate the bilayer thickness is to use the simulation box size according to the following equation:1
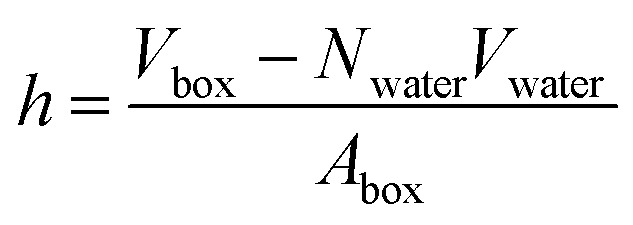
where, *h* is the thickness of the bilayer, *A*_box_ is the area of the bilayer, *V*_box_ is the total volume of the simulation box, *N*_water_ is the number of water molecules, and *V*_water_ is its respective volume.

There is direct relationship between area per lipid and membrane thickness. In fact, an increase in the average area per lipid is almost exactly compensated by a decrease in the lipid bilayer thickness. Also, disordered membranes and ordered membranes can be characterized according to membrane thickness and average area per lipid.

There are published reports showing that small molecules can reduce the membrane thickness. The thickness reduction should be large enough, where, generally, if the membrane thickness is slightly reduced, it is not enough to enhance solute permeation by reducing the span of the core hydrocarbon chain. However, it has been suggested that small solutes may move through the membrane by “hopping” between voids.^76^

If the change occurring in the membrane environment or inside the membrane results in an increase in the membrane thickness, the increase in thickness and decreased area per lipid are accompanied with higher ordering of the hydrocarbons of membrane, which can be traced using order parameters.

### 3.5 Membrane area compressibility

Compressibility is defined as a measure of the relative volume change of a given substance in response to stress. Thus, compressibility is extensively studied for lipid bilayers. Membrane area compressibility shows the resistance of the lipid bilayer to isotropic area dilation and is characterized by an area compressibility modulus, *K*_A_. The area compressibility modulus can be calculated using the following equation:2
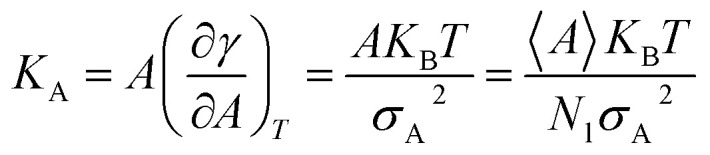
where, *γ* is the surface tension, *A* is the average total area, *σ*_A_^2^ is the mean square fluctuation, *k*_B_ is Boltzmann's constant, *T* is the temperature, 〈*A*〉 is the area per lipid (for pure membrane and/or mix lipid bilayers such as cholesterol–phospholipid bilayers), *N*_l_ is the number of lipid molecules (and if there is another molecule in the membrane such as cholesterol, the number of that molecule should also be added) and *σ*_A_ represents the standard deviation in the area per lipid. As can be concluded from eqn (2), the area compressibility modulus (and therefore the membrane compressibility and elasticity) is inversely proportional to area fluctuations. Thus, the area compressibility modulus, *K*_A_, is a good parameter for the rigidity of the lipid bilayer.

**Fig. 4:**
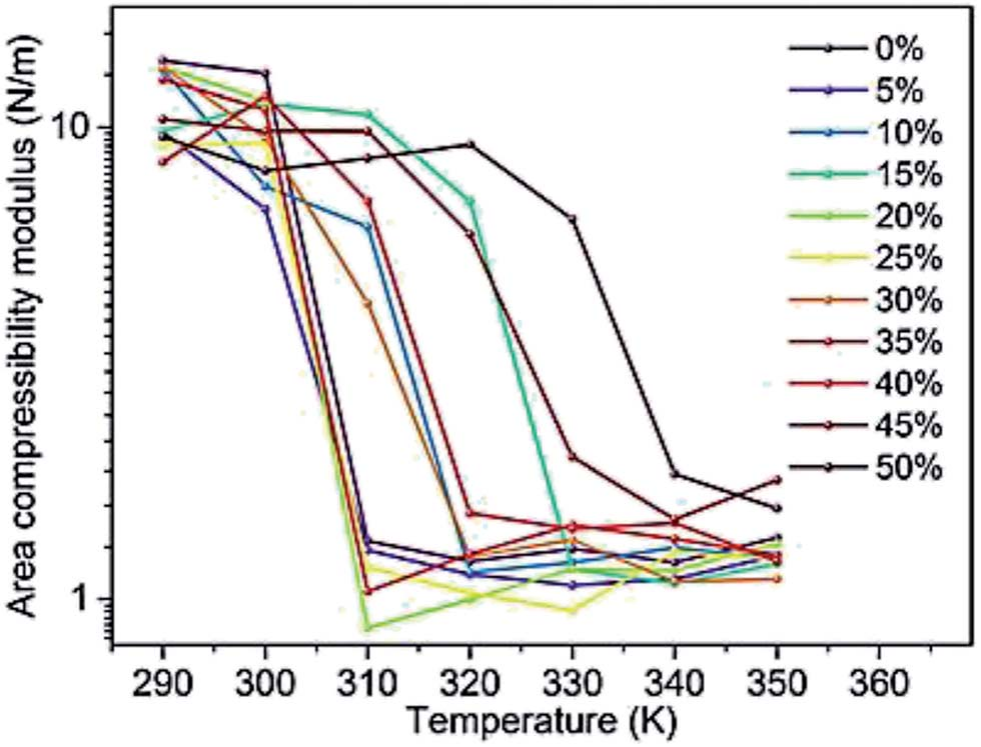
Area compressibility modulus as a function of temperature with the *y*-axis in the logarithmic scale. Reproduced from ref. 73 with permission from Elsevier.

Changes in the area compressibility modulus can be followed to follow membrane compressibility when a change occurs in membrane conditions. Wang and co-workers published a systematic study of the MARTINI coarse-grained model for a DPPC-cholesterol binary system.^77^ They studied *K*_A_ as a function of temperature (Fig. 4), and based on their results, the *K*_A_ values were used to determine the phase of the membrane (gel phase or liquid phase). For their systems, *K*_A_ values smaller than 2.1 N m^−1^ represent the liquid phase and *K*_A_ values larger than 4.2 N m^−1^ is for the gel phase.

### 3.6 Lateral diffusion

The lateral diffusion process in biological membranes has been investigated for several years. However, the exact mechanism of this type of movement is still vague and unclear, and efforts are continuing to further explore this issue.

**Fig. 5:**
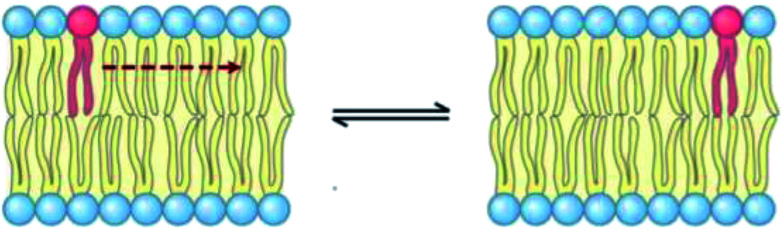
Schematic representation of lipid lateral movement.

The biological membrane behaves like a liquid and lateral diffusion refers to the lateral movement of its components, such as lipids and proteins, within each leaflet (Fig. 5). Basically, membrane components are generally free to move laterally if their mobility is not restricted due to their dependence on neighboring molecules. It should be noted that this type of movement is relatively fast and spontaneous. For the movement of lipid molecules (and other membrane molecules) the mean square of deviation is defined as follows:

3 MSD (*τ*) = 〈[*r⃑*(*τ*) − *r⃑*(0)]〉

where, *r⃑* is the position vector of a molecule and *τ* is the time step. Also, according to the Einstein diffusion equation, which can be used to describe the microscopic transport of molecules, the mean squared displacement (MSD) (*τ*) is proportional to the diffusion constant, *i.e.*,

4 MSD (*τ*) = 4*Dτ*

It should be noted that in the above equation, the factor 4 represents the diffusion in two dimensions.

Using eqn (1) and (2), it can be justified that the lateral diffusion coefficients can be computed using the following relation:^78^5
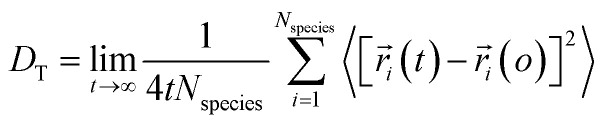


In the above equation, 
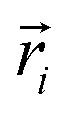
 is the center of mass (COM) position of molecule *i* at time *t*. Also, the sum is applied over all the molecules of a given species of the system. By tracking the position of each molecule in the upper leaflet of the membrane (or lower leaflet of the membrane), the lateral diffusion coefficients for each species can be calculated. It should be noted that this tracking of the position is carried out using the COM of each leaflet.

**Fig. 6:**
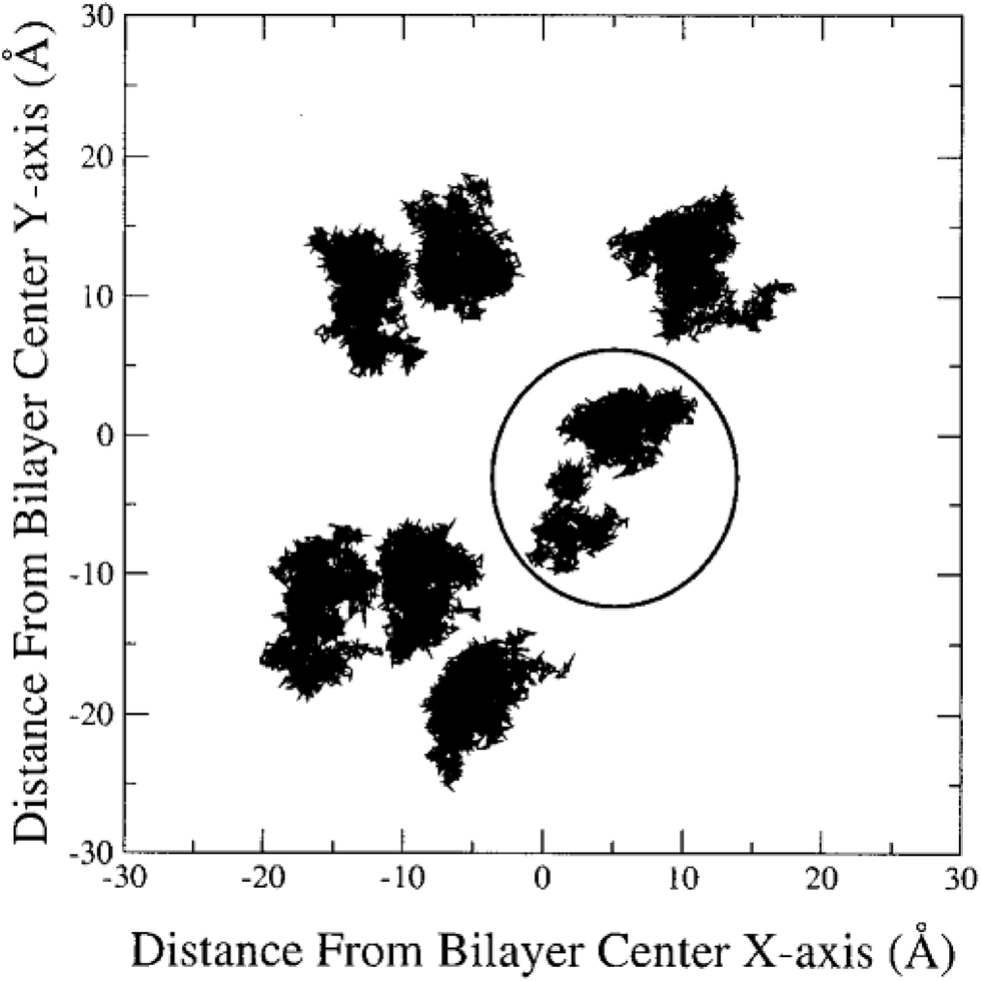
COM motion of selected DMPC projected onto the *XY* plane. The specified molecule with a circle has a “jump”. Reproduced from ref. 20 with permission from Elsevier.

In the literature, lateral diffusion in molecular dynamics simulation has been studied in some ways. In one way, a “qualitative picture” of the dynamics of molecular mobility can be presented. For example, Moore and coworkers published a study on a fully hydrated DMPC bilayer using molecular dynamics simulation.^20^ They considered seven molecules and the “stroboscopic” picture of the movements of COM of selected molecules projected onto the *XY*-plane was used (Fig. 6).

**Fig. 7:**
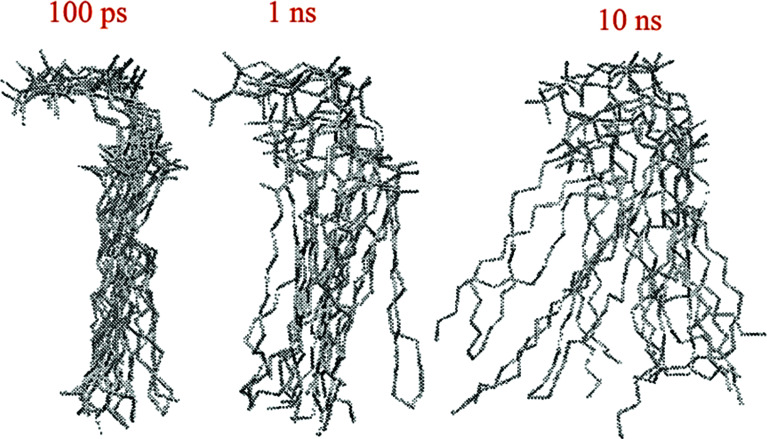
Ten superimposed structure snapshots for different time scales. Reproduced from ref. 20 with permission from Elsevier.

The other way to describe the dynamics of lipid molecules is superimposition of motions of a single lipid molecule on different time scales. For example, Moore used three different “time scales”: 100 ps, 1 ns, and 10 ns (ref. 20) (Fig. 7).

As can be seen, on the 100 ps time scale, the amount and amplitude of the movements are small and just intramolecular vibrations and a few torsional *gauche*–*trans* bond flips can be observed. On the 1 ns time scale, the amplitudes of the lipid motions increased and we see that the lipid has increased amplitude motion. A “smearing” can be observed in Fig. 7 in the 1 ns time scale, which is due to the translation and rotation of the lipid molecules and torsional *gauche*–*trans* flips in the tail region. However, the head group movements are very slow (due to the high interaction with each other and aqueous environment), and almost all the head groups are fixed in place and still oriented in roughly the same direction. In the time scale “10 ns”, as can be seen, lateral diffusion begins. In this time scale, large amplitude of motion of the tail and significant head group rotational motions can be seen.

**Fig. 8:**
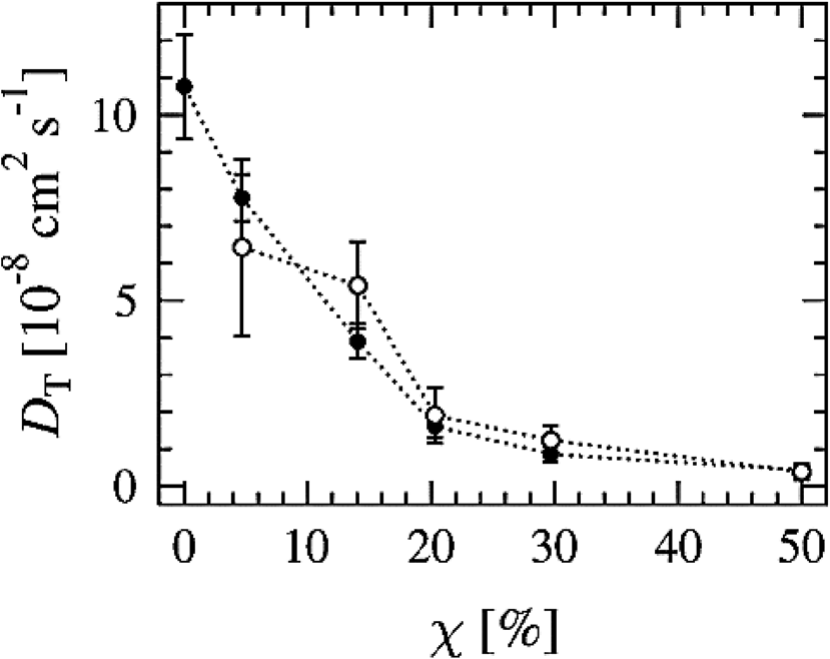
Lateral diffusion coefficients of DPPC (●) and cholesterol (○) molecules as a function of cholesterol concentration. Reproduced from ref. 74 with permission from Elsevier.

Another way to study lateral diffusion is following the lateral diffusion coefficient changes during the simulation. For example, Flack and coworkers reported the results for lateral diffusion coefficients changes in 100 ns molecular dynamics simulations to study the influence of cholesterol on the structural and dynamic properties of DPPC bilayers in the fluid phase (see Fig. 8).^78^ As can be seen, the lateral diffusion coefficients for both DPPC and cholesterol decreased monotonically with an increase in cholesterol content. It should be noted that the lateral diffusion can be evaluated only in the liquid crystal state and not in the gel or ripple phase.

Lateral diffusion is influenced by the temperature and increases with an increase in temperature.^77^

Another parameter that can be calculated is the rotational correlation function. In the literature, the rotational correlation function is defined as *C*(*τ*) = 〈*P*_2_(cos(*θ*(*τ*)))〉, where *θ*(*τ*) is the angle between the orientation vectors defined for the studied molecule such as lipid molecules separated by a time interval ‘*τ*’, *P*_2_ is the second Legendre polynomial, and 〈 〉 represents the ensemble average.^79–81^ Changes to this parameter can be followed during a simulation when applying a particular change and shown in a plot.

### 3.7 Rotational diffusion

**Fig. 9:**
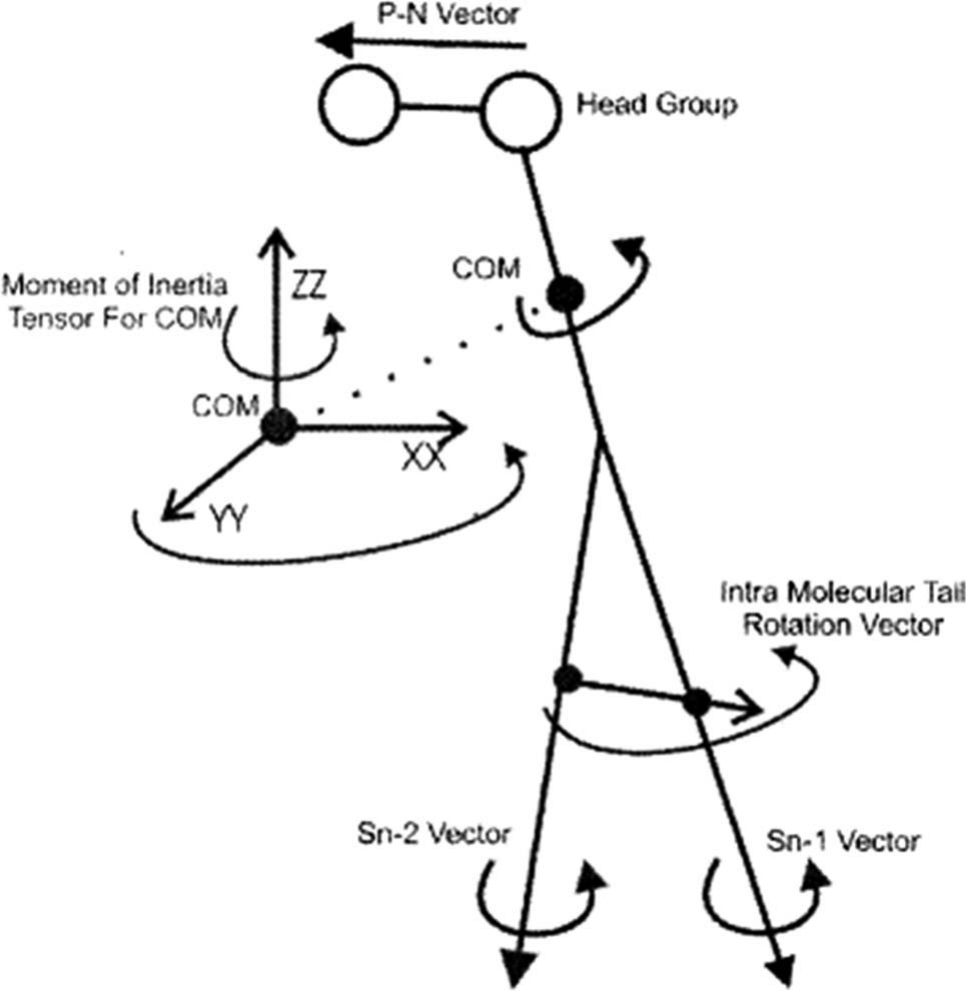
Illustration of the various vectors chosen to study the rotational dynamics of DMPC Moore and coworkers. Reproduced from ref. 20 with permission from Elsevier.

To study the rotational diffusion of a lipid molecule in a lipid bilayer, the first step is to define the main axis of rotation. The principal axis of rotation (or principal direction) is an eigenvector of the mass moment of inertia tensor defined relative to some point (typically the center of mass), which gives an idea of the overall rotation of the lipid molecule in the bilayer (Fig. 9).^20^ Other vectors can also be used to check the rotation of molecules. For example, a vector considered from the P atom to the N atom of the head group (the P–N vector) (see Fig. 9). This vector will provide useful information about the local environment at the lipid–water interface.

Other vectors can also be considered within a phospholipid to study the rotation of the molecule. For example, a vector can be considered from the center of mass of the Sn-1 tail to the center of mass of the Sn-2 tail. Also, vectors along the Sn-1 and Sn-2 chains can be defined from the first to the last carbon of each chain. The latter vectors can give data about the rotation properties inside the membrane.

The rotations of lipid molecules are characterized using the MSD (eqn (6)) of the various angular variables.^20^

6 MSD = (1/*n*)〈|*θ*_*i*_(*t*) − *θ*_*i*_(0)|^2^〉

In the above equation, the *θ* variable is defined as the angle between the projection of one of the vectors onto the *x*–*y* plane and the *x*-axes and *n* is the number of simulation steps. The *x*–*y* plane projection is used because it considers the rotational properties perpendicular to the bilayer normal. When checking *θ*, care must be taken that the variable samples the space from −∞ to +∞ and not from −π and +π (in radians) because this would give spurious results.^20^

To study the rotation diffusion, a parameter called rotational diffusion coefficient (*D*) (eqn (7)) is calculated and plotted *versus* time.

7 *D* = 〈|*θ*_*i*_(*t*) − *θ*_*i*_(0)|^2^〉

### 3.8 Order parameters

Based on their fluidity, biological membranes can be divided into three general categories: crystalline state, gel state and liquid state. Normally, in the crystalline state the lipid molecules are all ordered along their long axis in the same manner.^82^ When the membrane phase changes from the crystalline state to the gel state (*L*_β_), the average area per lipid increases, and the alkyl chains start to form gauche defects, the lipids are less mobile, more ordered, and closely packed and a reduction occurs in the system order. The gel to liquid crystalline phase (*L*_α_) transition is also accompanied by an increase in the surface area per lipid and a thinning of the bilayer due to the formation of extensive gauche defects. Thus, the high temperature *L*_α_ phase is characterized by considerable structural disorder.

One of the most common methods and perhaps the most common methods for characterizing the order in membranes is the use of order parameters (or lipid-tail-order parameters), which can be measured experimentally *via* deuterium NMR. In fact, order parameters provide a measure of the alignment of the hydrocarbon chains in the membrane. Specifically, order parameters give insight to the orientation of fatty lipid chains with respect to the bilayer normal. The order parameter (*S*_*z*_) can be defined for every C_*n*_ atom in the hydrocarbon chains as follows:8
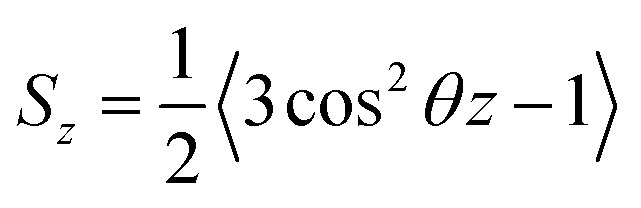
where, *θ*_*z*_ is the angle between the *z*-axis (the membrane normal) of the simulation box and the vector C_*n*−1_ to C_*n*+1_. In some studies, *θ* is considered as the time-dependent angle between a C–H bond along the acyl chain and the membrane plane normal (*z*-axis).^72^

In the above equation, the bracket denotes the time averaging over two bonds at each group of CH_2_ in all the lipids. If the united atoms force field is used in the simulation (which is more common), it should be to reconstruct the vector C_*n*−1_ to C_*n*+1_ from the positions of three successive CH_2_-groups assuming tetrahedral geometry of the CH_2_-groups.

It has been shown that the order parameters, *S*_*z*_, are correlated with some parameters of the membrane such as membrane rigidity and area expansion modulus as well as elastic properties, such as compressibility.^83^

The order parameter ranges from −0.5 to 1. A value of −0.5 implies that the two considered vectors are perpendicular to each other, whereas a value of 1 implies that the two considered vectors are parallel.

**Fig. 10:**
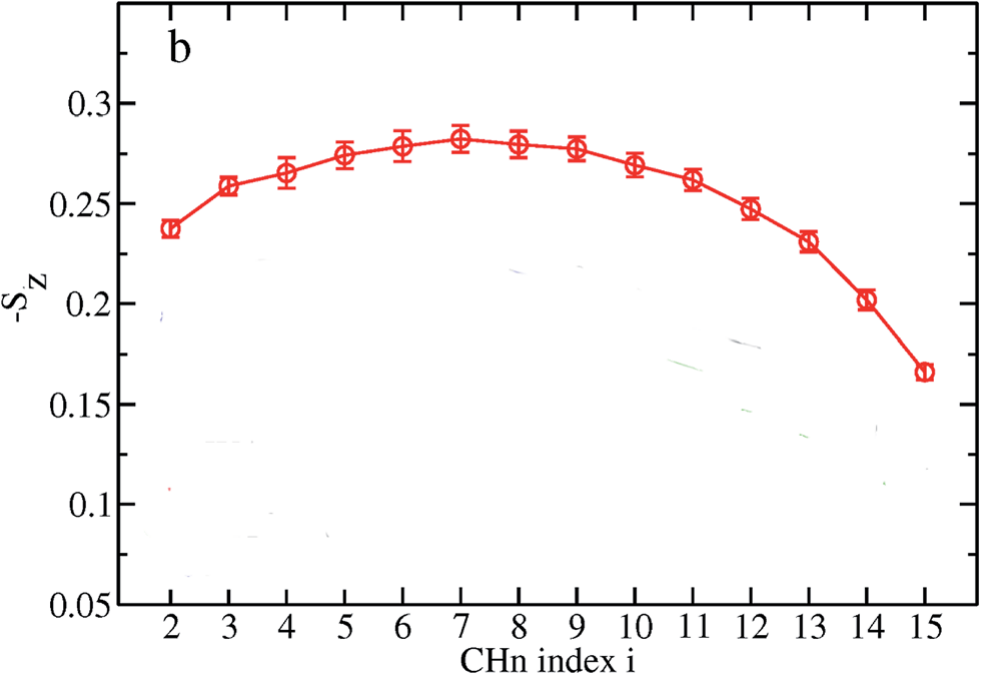
Order parameter profile for a typical molecular dynamics simulation.

The order parameter profiles for all membranes in a typical MD simulation are almost qualitatively similar. In a typical order parameter profile, a graph similar that in the Fig. 10 is obtained.

In this graph, there appears to be a higher degree of disorder in the alkyl groups close to the water interface, which is perhaps due to the interactions of the polar head groups of the lipid molecules with each other and with water molecules. The order of the tails then increases going down the chain and decreases again toward the center of the membrane. The disorder in the center of the membrane is because the chains have a greater degree of conformational flexibility, particularly in the longer chains, which extend into the core region. In this plot, if we compute the mean value, we actually calculate the average value of the parameter for all vectors passing through two carbon atoms.

The length of the hydrocarbon chain can affect the order parameters, where the longer chains reduce the order parameters. For example Notman and coworkers^84^ studied the order parameter profiles for a hydrocarbon chain with a length of 24 carbons and another with 16 carbons. As discussed, the order parameter profiles for the two studied systems were similar, with a lower overall ordering for the C24 chain relative to the C16 chain.

Also, temperature can affect the order parameters. The value of the order parameters of the hydrocarbon chains at lower temperatures are slightly more than that at higher temperature due to the closer packing of the lipids at a lower temperature.

Sometimes, there is a difference between the experimental parameter values and that obtained from molecular dynamics simulations, which is mainly attributed to the subtle defects in hydrocarbon chain potential, incomplete sampling of the chain conformations and/or long time-scale lipid stumble.^85^

### 3.9 Head group tilt

The head group tilt is an interesting parameter, which shows a characteristic tilt toward the bilayer normal. This parameter is obtained by calculating the distribution of the connecting vector of nitrogen and phosphorus atoms and then by averaging it over the simulation time for all lipids.^86^ It is known that many properties of biological membranes depend on the dipole moment associated with their zwitterionic head group (including nitrogen and phosphorus atoms), which are involved in long-range electrostatic interactions.^87^ To study the head group tilt in different conditions (such as in the presence of different molecules in the vicinity or inside the membrane or the application of different conditions to the membrane relative to biological conditions), its statistical distribution can be obtained and plotted relative to the bilayer normal during the simulation.^88^

### 3.10 Electron density profile

**Fig. 11:**
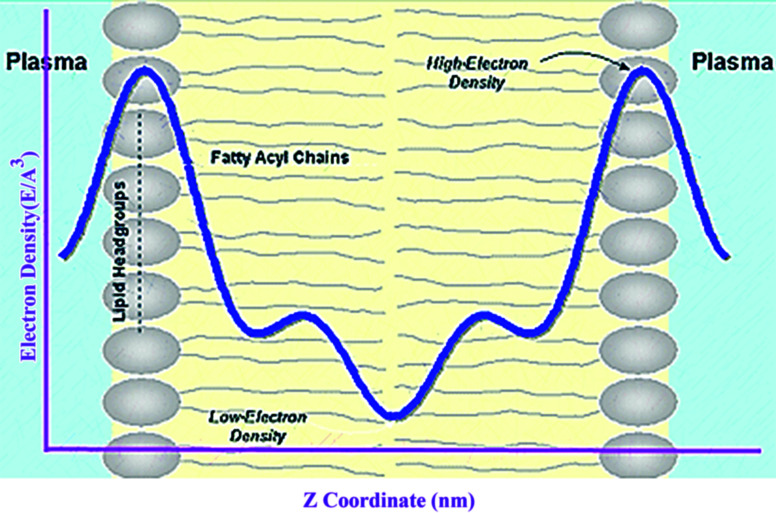
Electron density distributions along the bilayer normal.

Electron density can be defined as the measure of the probability of an electron being present at a specific location. Experimentally, the electron density of a membrane can be estimated using X-ray crystallography. In a typical electron density analysis, the time-averaged *z*-distributions of electrons in simulation is calculated, which can be evaluated with good accuracy since all the atomic positions are known. A typical electron density profile across the membrane is depicted in Fig. 11, which has the following characteristics: a minimum in the middle of the bilayer (near the CH_3_ groups), two maxima (peaks) at the position of the head groups (phosphate groups), and a local minimum in the water layer.^89^ If a polar molecule such as cholesterol is added to the membrane, a small shift of in the peaks associated with the phosphate groups occurs, while toward the bilayer center profile, either the membrane does not change or has a very slight change. It must be noted that the higher the concentration of the added molecule, the greater the change in the density profile of the membrane. Also, in addition to the change in the position of the peaks, the shape of the profile may change slightly toward the bilayer center compared to the pure membranes.^90^ In an electron density profile, the hydrophobic thickness is defined as the distance between the peaks of the electron density profile and changes may occur when a molecule is inserted into or interacts with the membrane.

When biological membranes are exposed to other molecules (either outside the membrane or inside the membrane) or when different conditions are applied to the membrane relative to physiological conditions, the overall shape of the profile, the location (shifting) and height of the peaks may change.^90^

### 3.11 Mass density profile

**Fig. 12:**
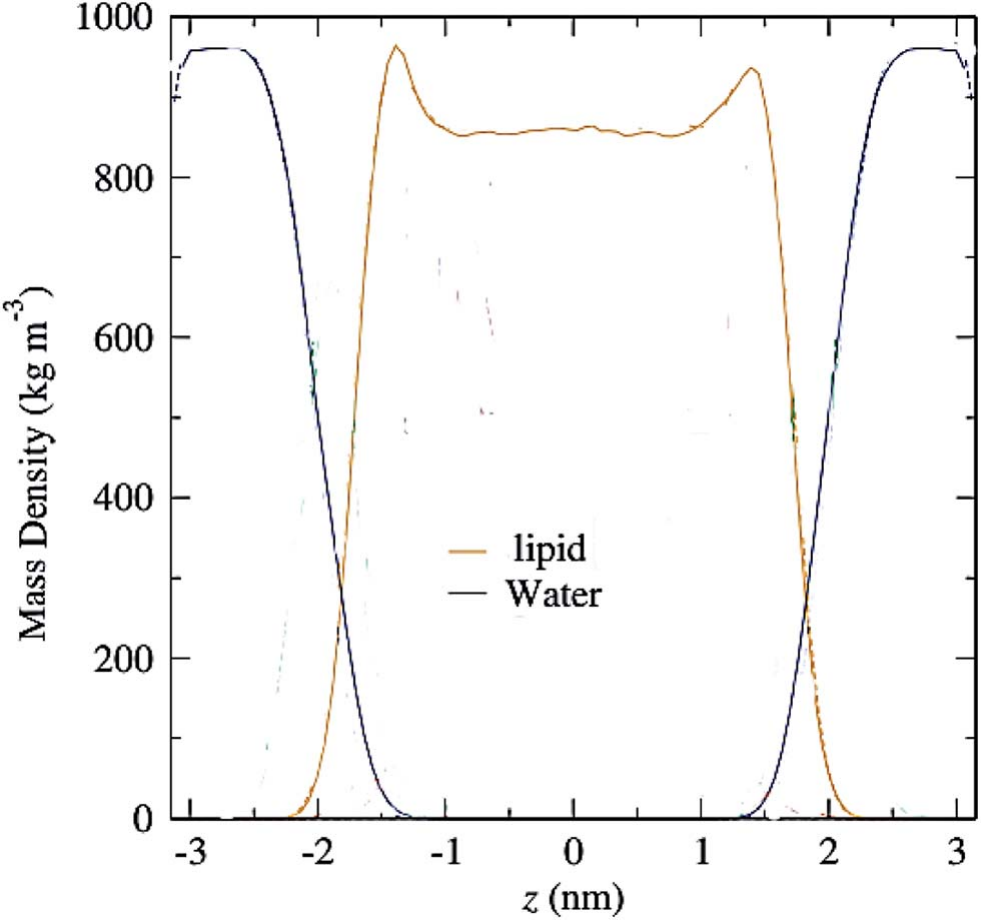
Mass density profiles of lipid and water molecules in a typical membrane. The center of the membrane is taken at *z* = 0.

Another parameter that can be calculated is the mass density profile across the bilayer, which shows how mass is distributed along the membrane *z*-axis. In a typical simulation, the mass density profile is the same shape as the electron density distribution (Fig. 12). For calculating the mass density profile and because the COM of membrane may fluctuate during the simulation, in each frame, the coordinates (*x*, *y*, *z*) of all the atoms are determined relative to the instantaneous COM (*z* = 0). The first step in the calculation is determining the coordinates of the center of mass of the membrane. This is calculated using the COM of the two leaflets. Then, for generating the mass density profile, the coordinates of all the atoms (including all hydrogen atoms) are considered with respect to the center.

According to the graph (Fig. 12), which is a mass density profile for a typical system including water and lipid, it is known that at the beginning of the lipid chain, the mass density is high and it simulates the mass density of several soft polymers (0.9–1.3 g cm^−3^). At carbon position 9 the density is reduced to the density of liquid hexadecane (0.753 g cm^−3^). In the middle of the membrane, the density is meaningfully lower, reduced to (0.60 g cm^−3^). From this it, can be concluded that the hydrocarbon interior of the bilayer in the liquid crystalline phase is far from homogeneous.

### 3.12 Electrostatic potential profile

The electrostatic asymmetry of bacterial outer membranes is of particular relevance for the development of novel antibacterial drugs since it affects the incorporation of membrane proteins into the membrane^4,5^ and acts as the driving force for antimicrobial peptide association with the membrane.^6–8^

The electrostatic potential profile of membranes is of particular relevance for the design and development of novel drugs (particularly drugs affecting the lipid bilayer and membrane proteins). This is because the distribution of electric charge affects how the proteins and lipid molecules are incorporated in the membrane^91,92^ and acts as the driving force for drug association with the lipid bilayer.^93,94^ In principle, calculating the electric charge distribution (electro-potential potential profiles) in a membrane is a seemingly straightforward and relatively simple task, which is calculated using Poisson's equation by integrating the charge density twice.^95^

The electrostatic potential profile across the membrane is calculated in a similar way as the mass density profile. The average charge density profile is first calculated in such a way that the center of the membrane (*z* = 0) is determined for each simulation frame separately. Finally, the electrostatic potential is determined by integrating the charge density twice starting from the initial condition *V* (*z* = 0) = 0.

### 3.13 Number of hydrogen bonds

The interactions between membrane lipids and water molecules at the lipid bilayer/water interfaces are responsible for many membrane function dynamics, stability of the biological membrane and are also strongly associated with numerous biological processes at the interfaces of lipid bilayers.

Due to the presence of polar headgroups in membrane lipids, they have the ability to form hydrogen bonds with each other and with molecules of water. In the presence of an interacting molecule or when applying different conditions than normal conditions (such as change, temperature, and ionic strength) this ability may change. Accordingly, a number of published studies have followed the changes in the number of hydrogen bonds and formation of different types of hydrogen bonds between interacting molecules and lipid molecules, interacting molecules and water molecules and lipids and water molecules during simulation.^88^ In these cases, a free membrane system in normal conditions is used as the control. If in the presence of the interacting molecule or when applying different conditions than normal conditions, the number of bonds increases, it shows that the penetration of water molecules into the membrane has increased, and thus the structure of the membrane has undergone a change. Also, increasing the number of hydrogen bonds between interacting molecules and the membrane indicates that these molecules are penetrated more into the membrane. It should be noted that for penetration through membranes, interacting molecules need to cross the charged lipid head groups, which are highly viscous, present an extensive network of hydrogen bonds and thus have low permeability. Therefore, molecular penetration into a membrane depends on the membrane thickness, type of polar head group, presence of pores and alternative mechanisms such as cyclic moieties or methyl branches to provide voids or pockets within the membrane that allow transportation.

### 3.14 Radial distribution function

Radial distribution functions (RDF) are one of the most important parameters that can be calculated for biological membrane systems using molecular dynamics simulation. Radial distribution functions can be calculated in different ways. One of the ways is that RDF is calculated around a (set of) atom(s). The other normal way is to calculate it around the center of a mass of a set of atoms or molecules.

Radial distribution function plots can be used to determine the hydration layers around the lipid bilayer. For example, Chiu and coworkers used a new equilibration procedure for the atomic level simulation of a hydrated lipid bilayer to hydrated bilayers of dioleylphosphatidylcholine (DOPC) and POPC.^57^ To determine the number of hydration layers, one way is that RDFs were calculated by simply finding the nearest lipid head group atom (phosphate oxygen) for each water molecule and then the resulting distribution of distances was binned. Their results showed that the peaks in DOPC and POPC RDFs are at the same radial locations. Also, their results confirmed that the main peak is sharper and higher for DOPC compared with POPC. Also, their RDF plots had two distinct peaks, which confirmed that the lipid molecules began to have two distinct hydration layers around them instead of just one. Another use of RDF plots is to prove the formation of hydrogen bonding.^88^ To do that, if the place where the first peak appears is less than a threshold for hydrogen bond formation (for example, hydrogen bonding distance of water lies in the interval of 0.163–0.199 nm (ref. 35)), it can be concluded that there is an H-bond between the water molecules and phosphate oxygen of the headgroup.^88^ Also, the height of the peak in the graph can present information about the density of molecules (or atoms). For example, if in the presence of a membrane interacting molecule, the height of first peak with respect to a reference peak increases, this can be considered as an increase in water penetration into the head groups.^88^ Peak height can also be a measure of the probability of hydrogen bond formation. The higher the peak, the more likely it is to form a hydrogen bond.

### 3.15 Head group dynamics

One of the other analysis that can be done on the head groups of lipid molecules is to determine their dynamics, which can be called “head group dynamics”. To calculate the head group dynamics, the velocity autocorrelation functions (VAFs) should be determined using the following equation:

9 *C*(*t*) = (〈*v*(0)*v*(*t*)〉)/(〈*v*(0)*v*(0)〉)

where, *v*(0) is the velocity of the head group at the first frame of MD and *v*(*t*) is the velocity at time *t*. The angular brackets in the above equation indicate averaging the velocities (*v*) over the entire MD trajectory. Also, the spectral density function *I*(*w*) is calculated using following equation:10
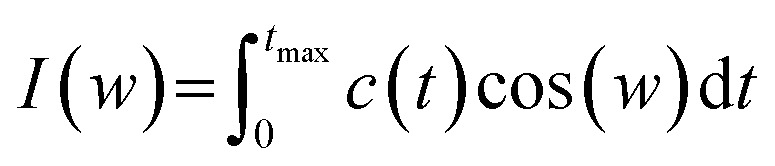


The head group dynamics depends on the degree of interactions between these groups with neighborhood groups, namely, head group-water and head group-head group interactions. As these interactions increase, the head group dynamics decreases.

There is a clear relationship between head group dynamics and the spectral density function. As far as the dynamics of the groups increase, *I*(*w*) is shifted to higher frequencies.

**Fig. 13:**
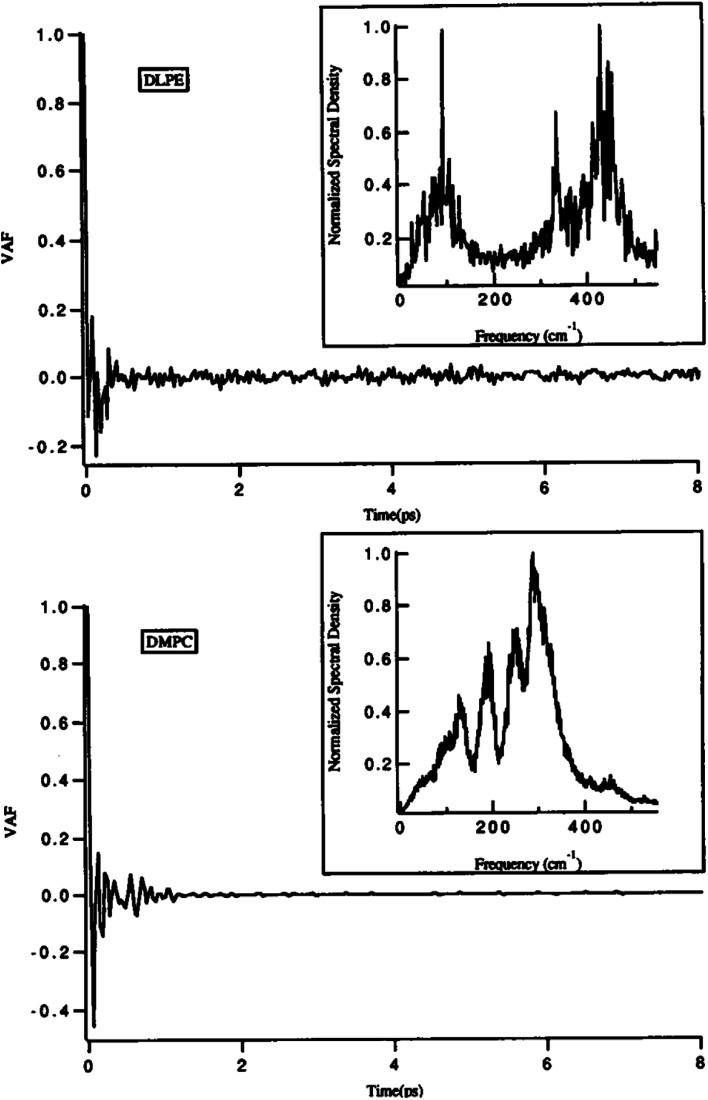
Velocity autocorrelation functions for the head groups of DLPE (top) and DMPC (bottom)-based bilayers. The normalized power spectra are shown as insets. Reproduced from ref. 89 with permission from Elsevier.

One of the main parameters that can affect the dynamics of the head groups is the formation of a hydrogen bond with the water molecules surrounding the lipid groups. For example, Damodaran and coworkers analyzed the head group dynamics by determining *c*(*t*) and *I*(*w*) for both a dilauroyl phosphatidylethanolamine (DLPE)-based bilayer and DMPC-based lipid bilayer^96^ (Fig. 13).

As it can be seen, the VAFs in Fig. 13 undoubtedly show the differences in head group dynamics. These differences are due to the differences in the amount and type of interactions the polar head group is involved with. In this study, the dilauroyl phosphatidyl ethanolamine head groups encountered a large number of collisions due to the frequent formation and rupturing of the hydrogen bonds with water and neighboring head groups. This caused the VAF to not decay smoothly to zero. Also, this resulted in *I*(*w*) shifting to higher frequencies for DMPC, which implies that the inter-head group and head group-solvent interactions are stronger for DLPE than DMPC.

If the VAF for a typical head group is decayed to zero rapidly, it implies a much smoother motion.^96^ Also, a higher VAF indicates that the head group is more conformationally flexible.

### 3.16 Local membrane curvature

The insertion of a protein or part of its domains into a lipid matrix will cause an increase in the surface area in one or both sides of the membrane. In the cases where the increment is not identical in the two bilayer leaflets, the differential increment in the membrane surface area may result in membrane local curvature. There are simple analytical theories based on the area difference between the two layers.DDA = DA outer − DA inner

Small differential increments of surface area may indicate extreme membrane curvature.^97^

## 4 Conclusion

Presently, with the significant advances in computer hardware technology and advancement in algorithms for MD simulation, more biological systems with a longer timescale can be studied. However, these improvements are still insufficient to investigate phenomena on an appropriate timescale. In particular, this is true of the phenomena involved in lipid bilayers. Even if different lipid bilayer phenomena can be studied at the appropriate time scale, the important challenge faced is how to analyze this great amount of data.

This review gave an overview and discussion of the some of the most important possible analysis technical challenges, and existing protocols that can be performed on the biological membrane by molecular dynamics simulation.

The reviewed analyses included degree of membrane disruption, average area per lipid, probability distributions for the area per lipid molecule, membrane thickness, membrane area compressibility, lateral diffusion, rotational diffusion, order parameters, head group tilt, electron density profile, mass density profile, electrostatic potential profile, ordering of vicinity waters, number of hydrogen bonds, radial distribution function, and head group dynamics.

Regarding all of these possible analyses, it seems that MD simulation is capable of presenting structural and dynamic structural data from the lipid bilayer.

## Conflicts of interest

There is no declaration of interest.

## Supplementary Material
