# Correction: Shedding light on the structural properties of lipid bilayers using molecular dynamics simulation: a review study

**DOI:** 10.1039/c9ra90014d

**Published:** 2019-03-12

**Authors:** Sajad Moradi, Amin Nowroozi, Mohsen Shahlaei

**Affiliations:** Nano Drug Delivery Research Center, Kermanshah University of Medical Sciences Kermanshah Iran; Pharmaceutical Sciences Research Center, Faculty of Pharmacy, Kermanshah University of Medical Sciences Kermanshah Iran; Medical Biology Research Center, Kermanshah University of Medical Sciences Kermanshah Iran mohsenshahlaei@yahoo.com

## Abstract

Correction for ‘Shedding light on the structural properties of lipid bilayers using molecular dynamics simulation: a review study’ by Sajad Moradi *et al.*, *RSC Adv.*, 2019, **9**, 4644–4658.

The authors regret that incorrect details were given for ref. 22, 26 and 81 in the original article. The correct versions of ref. 22, 26 and 81 are given below.

The Royal Society of Chemistry apologises for these errors and any consequent inconvenience to authors and readers.

## Supplementary Material
